# Short and Long-Term Outcomes of Epidural or Intravenous Analgesia after Esophagectomy: A Propensity-Matched Cohort Study

**DOI:** 10.1371/journal.pone.0154380

**Published:** 2016-04-25

**Authors:** Wei Li, Yongchun Li, Qingyuan Huang, Shengquan Ye, Tiehua Rong

**Affiliations:** 1 Sun Yat-Sen University Cancer Center; State Key Laboratory of Oncology in South China; Collaborative Innovation Center for Cancer Medicine, Guangzhou, China; 2 Shanghai Chest Hospital, Shanghai Jiao Tong University, Shanghai, China; Baylor College of Medicine, UNITED STATES

## Abstract

**Background and Objectives:**

As a well-established technique for postoperative pain relief, the benefits of epidural analgesia (EDA) have been under debate recently. This study aimed to determine whether EDA could improve perioperative outcomes and survival in patients undergoing esophagectomy.

**Methods:**

From January 2010 to December 2012, 587 consecutive cases undergoing McKeown-type esohpageactomy were retrospectively identified from a prospectively maintained database.

**Results:**

After propensity-matching, incorporating baseline characteristics, 178 cases were included in each group, and patients characteristics distributions were well-balanced between two groups. Compared with intravenous analgesia, the use of EDA significantly decreased the incidence of pneumonia from 32% to 19.7% (*P* = 0.008), and anastomotic leakage from 23.0% to 14.0% (*P* = 0.029). The change in CRP level of EDA group was significantly decreased (preoperative, 6.2 vs. 6.2; POD 1, 108.1 vs. 121.3; POD 3, 131.5 vs. 137.8; POD 7, 69.3 vs. 82.1 mg/L; *P* = 0.044). EDA patients had a significantly longer duration of indwelling urinary catheter (*P*<0.001), and lower levels in both systolic (*P* = 0.001) and diastolic blood pressure (*P*<0.001). There weren't significant differences in overall survival (log-rank *P* = 0.47) and recurrence (Gray-test *P* = 0.46) between two groups.

**Conclusions:**

These findings revealed that EDA could attenuate inflammatory response and reduce the incidence of pneumonia and anastomotic leakage after esophagectomy, at the price of delayed urinary catheter removal and lower blood pressure. EDA remains an important component of multimodal perioperative management after esophagectomy.

## Introduction

Epidural analgesia (EDA) is a well-established technique for postoperative pain management that has been widely used for decades. The advantages of EDA, such as better postoperative pain relief and improved perioperative outcomes, once led EDA to be considered as the gold standard for pain management after major surgery. It has been suggested that the use of EDA ameliorates perioperative immune suppression, and reduced risk of recurrence and extended survival have been demonstrated subsequently among patients with breast[1] or prostate cancer[2]. With the huge evolution in perioperative management, however, some new evidence suggests that the benefits of EDA are not as significant as previously thought, especially in less invasive operations[3, 4]. The protective effects of EDA have been under great debate recently, and the use of EDA has shown a tendency of continuous decrease[5].

Mckeown-type esophagectomy, one of the most common procedures for nonmetastatic esophageal cancer, is a cervicothoracoabdominal procedure and is also one of the most invasive operations with high postoperative morbidity and mortality[6, 7]. Pulmonary complications and anastomotic leakage are the most common serious morbidities, and are great challenges to surgeons, even in experienced centers [8]. Major complications contribute to substantial perioperative mortality, and dramatically deteriorate quality of life[9, 10]. The importance of minimizing the risk of surgical complications can never be overstated. The investigations on the effects of EDA on postoperative outcomes for patients undergoing esophagectomy have been limited so far.

To test the hypothesis that EDA could inhibit postoperative inflammatory response, and improve perioperative outcomes and survival when compared with intravenous analgesia (IVA) in patients undergoing major surgery (esophagectomy), we performed the present study, by employing a prospectively maintained esophageal cancer database and conducting propensity matching to compensate for the differences in baseline characteristics.

## Population and Methods

### Ethics statement

All study protocols were approved by the Institutional Review Board of Sun Yat-Sen University Cancer Center. Written informed consent was obtained from each patient. All patient data were anonymized and de-identified in a confidential manner.

### Study population

Retrospectively screening a prospectively maintained esophageal cancer database which was constructed in 2014, a total of 587 consecutive cases undergoing elective McKeown-type esohpageactomy from January 2010 to December 2012 in Sun Yat-Sen University Cancer Center (Guangzhou, China) were identified. Among them, 543 cases receiving epidural or intravenous analgesia were eligible for this study. The database recorded information regarding sociodemographic data, treatment administration, perioperative parameters and follow-up status. Tumors were staged according to the 7th edition of the AJCC staging system[11]. The Mckeown-type procedure was described previously[10, 12]: first involved esophageal mobilization and radical mediastinal lymphadectomy through right thoracotomy or thoracoscopy, and then laparotomy or laparoscopic gastric mobilization. A gastric tube is fashioned with a width of approximately 4–5 cm, and pulled through retrosternal or posterior mediastinal routes to the left neck. A mechanical circular stapler was used to complete the esophagogastric anastomosis.

### Anesthesia and analgesia techniques

Induction of anesthesia was performed with midazolam (0.03–0.05mg/kg), propofol (1–2 mg/kg) or etomidate (0.3–0.5 mg/kg), sufentanil (0.3–0.5 μg/kg) or fentanyl (0.003–0.005 mg/kg), and cisatracurium (0.2–0.3 mg/kg). After tracheal intubation, all patients received balanced general anesthesia, which was maintained with sevoflurane (2%-4%, Mac value0.7–1.5) in 100% oxygen or propofol (6–8 mg/kg/h), followed by remifentanil (0.1–0.3 ug/kg/min) and cisatracurium (0.1–0.15 mg/kg/h). Except for opioids used at induction of anesthesia and postoperative analgesia, no additional opioids were administered during the maintenance of anesthesia, but non-steroidal anti-inflammatory drugs (flurbiprofen or parecoxib) were always used.

For patients in the EDA group, epidural catheter was inserted at thoracic 6–10 level before induction of general anesthesia. A test dose of of 3–5 mL of 1% lidocaine was administered as soon as the epidural catheter was in place, a mixture of 0.2% ropivacaine and mophine 0.02 mg/ml was injected epidurally as a loading dose before the end of the operation, then a continuous perfusion of 0.125% ropivacaine with morphine 0.06–0.1 mg/ml were administered through the epidural catheter at a rate of 2 mL/h until 48 h after surgical procedure. For the IVA group, the methods varied with the preference of anesthesiologists. The most common solution was a mixture of sufentanil (1–1.5 ug/ml) plus flurbiprofen (1–1.5 mg/mL), 10–15 ug sufentainl plus 50 mg flurbiprofen as a loading dose before the end of the operation; less frequently used solutions were mixtures of fentanyl (0.008–0.012 mg/ml) and flurbiprofen (1–1.5 mg/ml), 0.1–0.2 mg fentanyl and 50 mg fluriprofen as a loading dose; both were administered by intravenous at a rate of 2 mL/h until 48 h after surgical procedure. Patient-controlled technique wasn't routinely used.

### Perioperative management and follow-up

The preoperative workup regularly included physical examination, complete blood cell count, serum biochemistry tests, computed tomography, endoscopic ultrasonography, pulmonary function test and histological diagnosis. Surgical techniques have been described previously[12]. Normally, blood cell count, serum biochemistry tests were performed in postoperative day (POD) 1, 3 and 7, and temperature, heart rate and blood pressure were measured at 7 a.m. everyday.

Pneumonia was defined as clinical manifestation of pneumonia or bronchopneumonia confirmed by a new or progressive infiltrate on chest radiography and a positive sputum culture during postoperative hospital stay[9]. Anastomotic leakage was defined liberally by any extravasation of water-soluble contrast during swallow study, visualization of anastomotic dehiscence or fistulae during endoscopy or visible loss of saliva through the cervical wound[10].

The follow-up protocol was in accordance with our previous studies[13], and performed by the oncologic outpatient clinic or official contact with patients or their relatives by telephone. The last follow-up was April 30^th^, 2015, and the median follow-up was 34.9 (interquartile range, 17.7–43.5) months.

### Statistical analysis

Categorical variables were expressed as percentage, and compared using χ^2^ test. All imbalanced variables with a significance level of *P*<0.10 on χ^2^ test between two groups were included in the logistic regression to calculate the propensity score, modelling the probability of a patient receiving EDA. A one-to-one match without replacement was performed by using nearest-neighbor matching method with a caliper of 0.02 (0.2 of standard deviation) as recommended by Dr. Austin[14]. Normal continuous variables were expressed as mean ± standard deviation and compared by *t* test, and nonnormal ones as expressed as median (range) and compared by Mann-Whitney U test. Two-way repeated-measures analysis of variance (ANOVA) was applied to compare changes in the parameters of two groups.

Overall survival was analyzed with Kaplan-Meier curves and log-rank test. The risk of recurrence (defined as the cumulative incidence of recurrence [CIR]) was estimated using a cumulative incidence function, which accounted for death without recurrence as a competing event[15]. Differences in CIR between groups were assessed using the methods by Gray[16]. Statistical analyses were undertaken using SPSS 22.0 software for windows (SPSS Inc., Chicago, IL, USA) and R version 3.1.0 (http://www.r-project.org/). Statistical significance was set at *p*<0.05 and all tests were two-sided.

## Results

### Baseline patient characteristics

**Fig 1:**
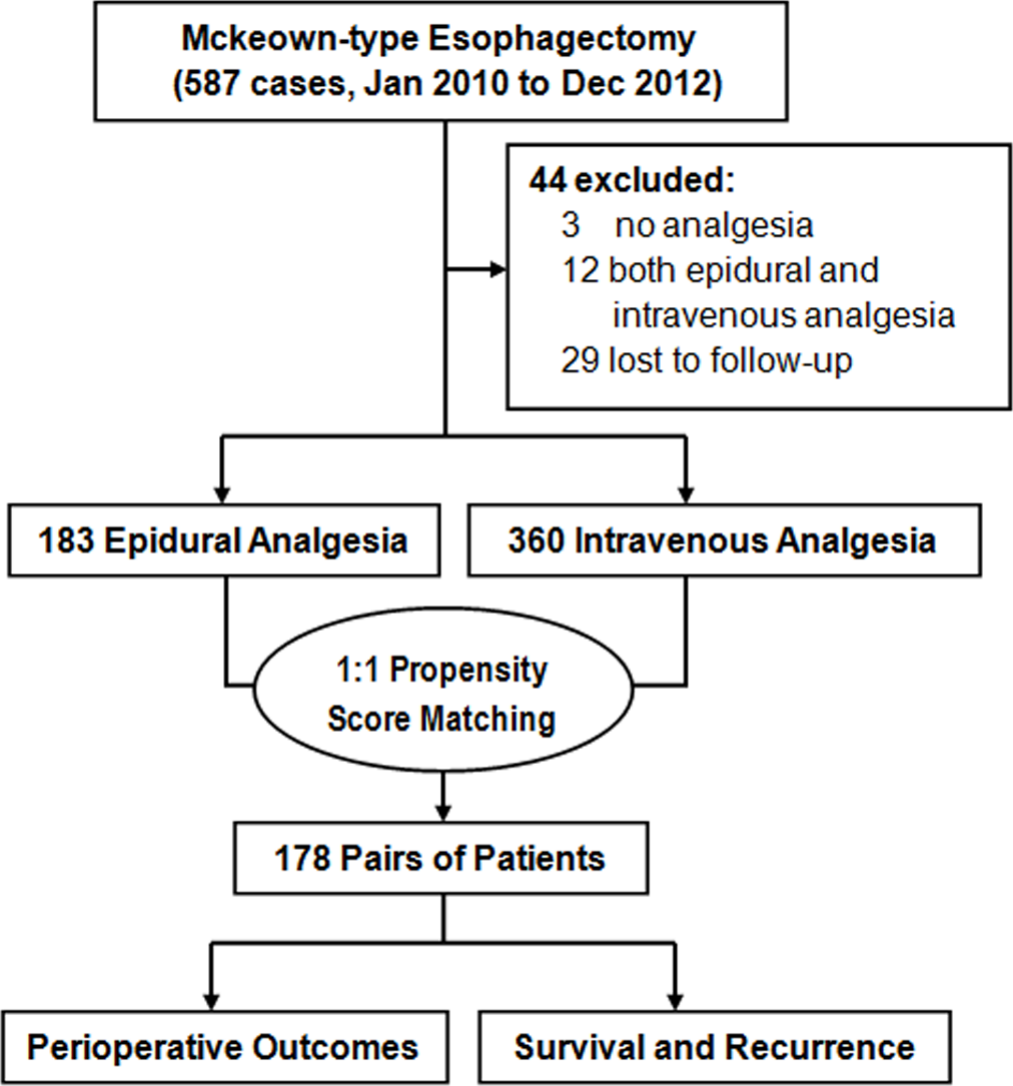
The selection and matching process of participants.

The selection and matching process of participants was showed in Fig 1. Among 543 eligible patients, 183 patients received EDA successfully, and the other 360 ones received IVA. Patients in EDA group were more likely to be male (*P* = 0.012) and undergo open esophagectomy (*P*<0.001). There was a trend that EDA group had higher proportions of normal preoperative percentage of predicted forced expiratory volume in 1 second (FEV1%) (P = 0.062) and C-reactive protein (CRP) level (*P* = 0.053). Propensity score were estimated by the above mentioned parameters, and matching based on similar scores produced 178 patients in each group. The distributions of patients' characteristics were well-balanced between EDA and IVA groups after matching. The baseline characteristics of two groups, before and after matching, were summarized in Table 1.

**Table 1:** Distribution of patients characteristics of epidural and intravenous analgesia groups, before and after propensity score matching.

Characteristics	Before Matching			After Matching		
	EDA (%)	IVA (%)	*P* value^#^	EDA (%)	IVA (%)	*P* value^#^
**Total**	183	360		178	178	
**Male gender**	153(83.6)	271(75.3)	**0.012**	151(84.8)	151(84.8)	1.00
**Age** ≤ 60 yr	102(55.7)	191(53.1)	0.51	100(56.2)	101(56.7)	0.95
**Smoking, never**	75(41.0)	108(59.0)	0.48	70(39.3)	71(39.9)	0.91
** >20 pack-years**	89(48.6)	164(45.6)	0.56	87(48.9)	93(52.2)	0.60
**Never drinker**	149(81.4)	282(78.3)	0.40	144(80.9)	139(78.1)	0.51
**FEV1%**≥80%	119(65.5)	262(72.8)	0.062	119(66.9)	119(66.9)	1.00
**DLCO%**≥80%	151(82.5)	279(77.5)	0.18	147(82.6)	134(75.3)	0.12
**Preoperative CRP** < 10 mg/L	163(89.1)	298(82.8)	0.053	158(88.8)	158(88.8)	1.00
**Comorbidity No.**			0.20			0.62
0	144(78.7)	265(73.6)		139(78.1)	135(75.8)	
≥1	39(21.3)	95(26.4)		39(21.9)	43(24.2)	
**ASA score**			0.41			0.23
1	10(5.5)	35(9.7)		9(5.1)	20(11.2)	
2	158(86.3)	289(80.3)		155(87.1)	144(80.9)	
3	15(8.2)	36(10.0)		14(7.9)	14(7.9)	
**Anesthesia duration**>480 min	101(55.2)	176(48.9)	0.17	98(55.1)	80(44.9)	0.11
**Surgical type**			**<0.001**			1.00
Open	142(80.2)	209(60.4)		137(77.0)	137(77.0)	
MIE	35(19.8)	137(39.6)		41(23.0)	41(23.0)	
**Pathologic stage**			0.84			0.76
0	5(2.7)	6(1.7)		5(2.8)	4(2.2)	
I	18(9.8)	33(9.2)		17(9.6)	13(7.3)	
II	71(38.8)	148(41.1)		68(38.2)	71(39.9)	
III	88(48.1)	167(46.4)		87(48.9)	87(48.9)	
IV	1(0.7)	6(1.7)		1(0.6)	3(1.9)	
**Median follow-up,** (months)	36.7	32.5	0.02*	35.8	33.9	0.10*

# χ^2^-test or Fisher’s exact test

* Mann-Whitney U test. *P*<0.05 was highlighted in **bold.**

Abbreviations: EDA, epidural analgesia; IVA, intravenous analgesia; FEV1%, percentage of predicted forced expiratory volume in 1 second; DLCO%, diffusing capacity for carbon monoxide expressed as a percentage of predicted; CRP, C-reactive protein; ASA, American Society of Anesthesiologists; MIE, minimally invasive esophagectomy.

### Short-term outcomes

Table 2 summarized the perioperative outcomes between the two groups. Among 178 included patients in each group after matching, 35 patients (19.7%) in EDA group and 57 (32%) in IVA group developed pneumonia (*P* = 0.008). EDA also reduced the incidence of acute respiratory distress syndrome (ARDS), although it didn't reach statistical significance (*P* = 0.082). The incidence of anastomotic leakage in EDA group was 14.0%, and was significantly lower than that of IVA group (23.0%, *P* = 0.029).

**Table 2:** Perioperative outcomes of epidural and intravenous analgesia groups.

Outcomes	EDA	IVA	*P* value
**Pneumonia**	35(19.7)	57(32.0)	**0.008**
**ARDS**	5(2.8)	12(6.7)	0.082
**Anastomotic Leakage**	25(14.0)	41(23.0)	**0.029**
**Readmission to ICU**	10(5.6)	18(10.1)	0.12
**ICU stay (d)**^#^	1.5(0–31)	1(0–65)	0.063
**Postoperative hospital stay (d)**^#^	17(8–504)	18(5–210)	0.39
**Indwelling urinary catheter (d)**^#^	3(2–40)	3(2–27)	**<0.001**
**In-hospital mortality**	2(1.1)	2(1.1)	1.00

Data are frequency (percentage) or median (range).

# Skewed distribution, Mann-Whitney U test applied. *P*<0.05 was highlighted in **bold.**

Abbreviations: EDA, epidural analgesia; IVA, intravenous analgesia; ARDS, acute respiratory distress syndrome.

The median ICU stay was 1.5 days (range, 0–31 days) for EDA patients, and 1 day (range, 0–65 days) for IVA patients (*P* = 0.063). EDA patients had a significantly longer duration of indwelling urinary catheter (median, 3 days; range, 2–40 days) than that of IVA patients(median, 3 days; range, 2–27 days; *P*<0.001). Readmission to ICU, postoperative hospital stay and in-hospital mortality didn't differ significantly between the groups.

**Fig 2:**
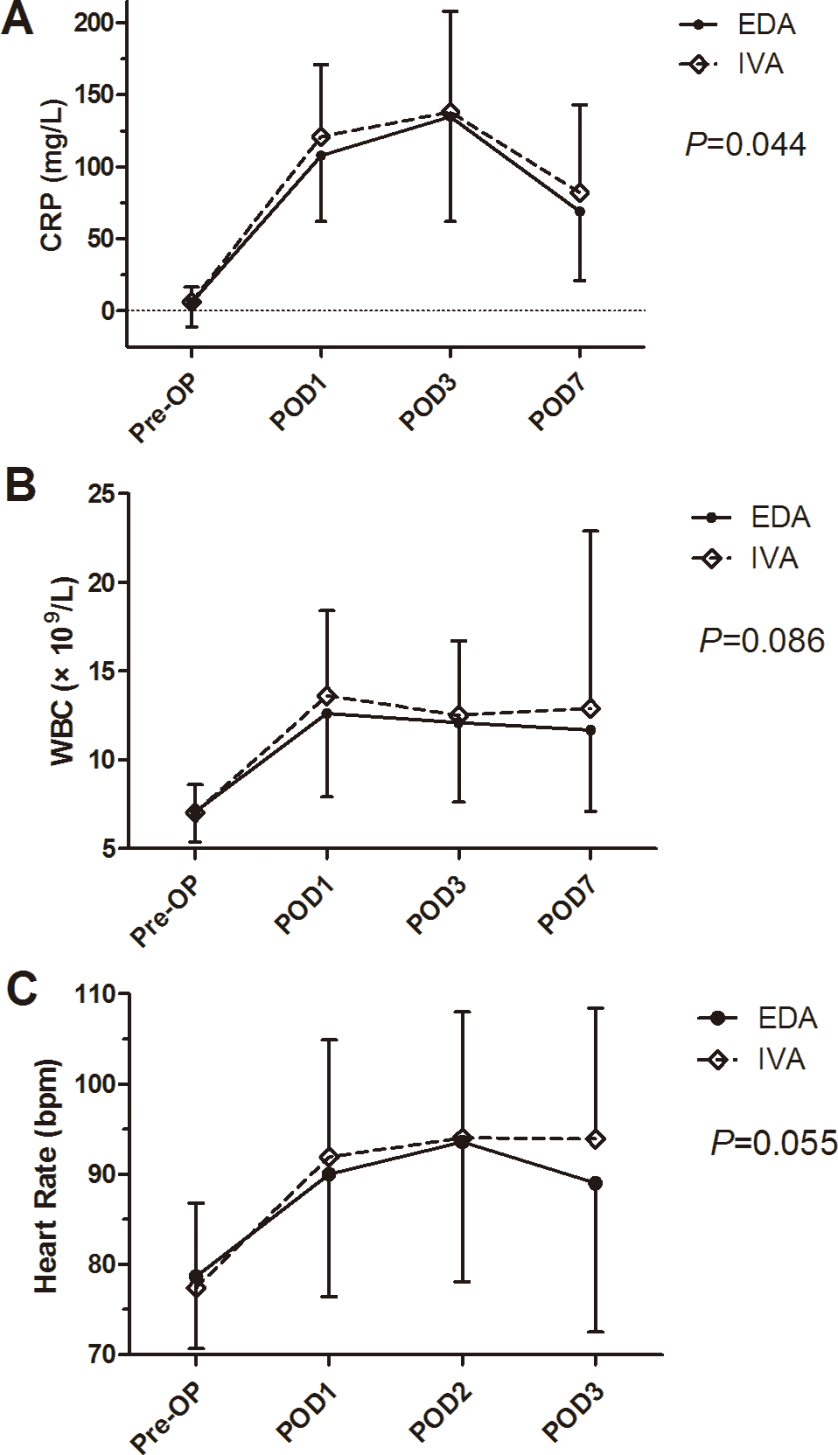
**The preoperative and postoperative changes of levels in C-reactive protein (A), white blood cell (B) and heart rate (C) between epidural analgesia (EDA) and intravenous analgesia (IVA) groups.**

**Fig 3:**
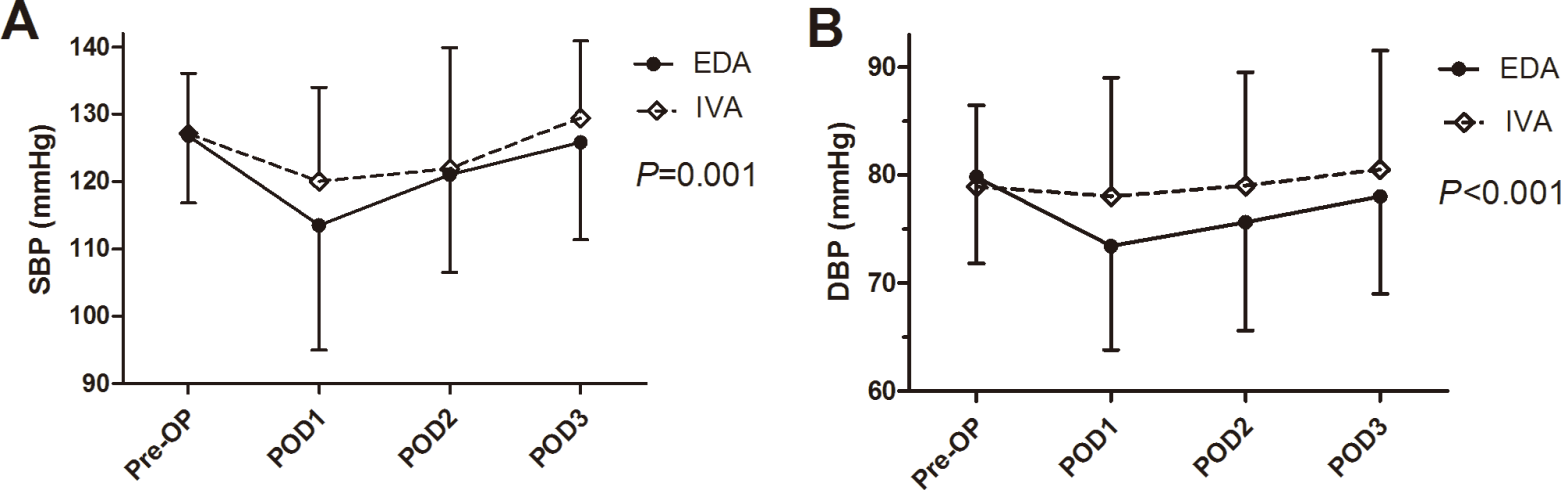
**The preoperative and postoperative changes of levels in systolic (A) and diastolic (B) blood pressure between epidural analgesia (EDA) and intravenous analgesia (IVA) groups.**

The CRP levels in two groups climbed dramatically after operation, and peaked in POD 3. The change in CRP of EDA group was significantly lower than that of IVA group (preoperative, 6.2 vs. 6.2; POD 1, 108.1 vs. 121.3; POD 3, 131.5 vs. 137.8; POD 7, 69.3 vs. 82.1 mg/L; *P* = 0.044, Fig 2A). There were marginally significantly lower levels of white blood cell (*P* = 0.086, Fig 2B) and heart rate (*P* = 0.055, Fig 2C) in EDA group. Comparisons of changes in mean systolic blood pressure (SBP, *P* = 0.001, Fig 3A) and diastolic blood pressure (DBP, *P*<0.001, Fig 3B) revealed significantly lower levels in EDA patients.

### Survival and disease recurrence

**Fig 4:**
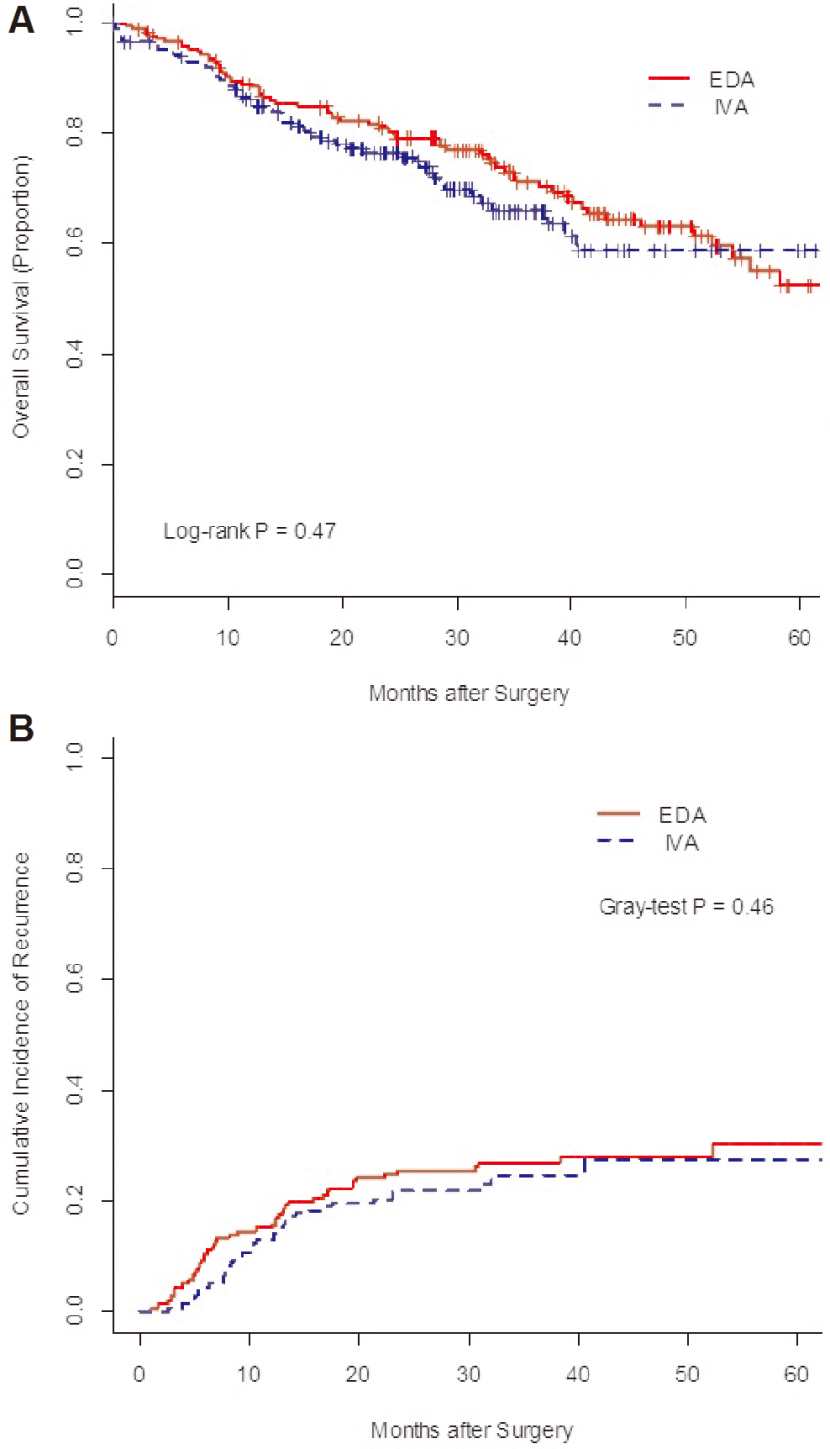
**Overall survival (A) and cumulative incidence of recurrence (B) of patients with esophageal cancer between epidural analgesia (EDA) and intravenous analgesia (IVA) groups.**

The 3-year overall survival of EDA and IVA patients were 70.8% (95% confidence interval[CI], 63.5–78.1%) and 67.6% (95% CI, 59.2–75.6%, log-rank *P* = 0.47, Fig 4A). The 3-year cumulative incidence of recurrence (CIR) was 26.9% (95% CI, 20.1–33.7%) and 24.5% (95% CI, 17.3–31.8%) of EDA and IVA groups, respectively (Gray-test *P* = 0.46, Fig 4B).

## Discussion

Esophageal cancer is one of the most common malignancies worldwide, and the incidence of adenocarcinoma has been dramatically increasing in western countries [17]. It is of huge importance to determine the optimal postoperative pain management for esophagectomy, a highly invasive procedure. Although it has been established that EDA provides better acute pain control and perioperative pathophysiology, whether these benefits after esophagectomy could be translated to improvements in short and long-term clinical outcomes has been scantly investigated. Previous studies yielded conflicting results, and were habitually limited by small sample sizes[18–20]. The present large-scale propensity-matched analysis reveals that the benefits EDA include attenuated inflammation, reduced risk of pulmonary complications and anastomotic leakage, although it also delays urinary catheter removal and lowers postoperative blood pressure transiently.

The current anesthetic practice in esophagectomy is one lung ventilation (OLV) to facilitate surgical exposure. The ischemia/reperfusion of the ipsilateral lung and high oxygen concentrations of the ventilated lung could trigger an inflammatory response [21]. Systemic inflammatory response plays a crucial role on the postoperative pathophysiological changes, and may result in acute lung injury [22]. Besides, the discharge of sputum after esophagectomy is usually insufficient due to severe wound pain. Collectively, major respiratory complications can reach above 30%, even in experienced centers[8]. The use of EDA provides excellent solutions to these problems. First of all, the lower levels of CRP, white blood cell and heart rate in EDA patients indicate that EDA could attenuate the inflammatory response after esophagectomy, in accordance with the results of various operations[23, 24]. In addition, the outstanding control of acute pain after esophagectomy[25] help patients to cough vigorously and discharge sputum timely and sufficiently, preventing pulmonary complications.

Another important finding of current study is the protective effects of EDA on anastomotic leakage, and it could be easily explained. Ischemia of gastric conduit[26] and impairment in oxygen supply [27] are the most important predisposing factors for leakage, besides anastomotic techniques. Michelet *et al* [28] used a laser Doppler flowmeter to measure the gastric mucosal blood flow of patient undergoing esophagectomy, and found that EDA improved the microcirculation of the gastric tube. In addition, EDA reduces the incidence of pulmonary complications, maintains adequate oxygen delivery postoperatively, and consequently promotes the tissue healing of anastomosis.

These findings have significant research implications as well. Current clinical investigations and trials on the perioperative outcomes after esophagectomy may neglect the effects of analgesia methods sometimes. Future such studies should obtain detailed data on analgesia method, consider it as a potential confounding factor, and adjust for or stratify by it during analysis.

The favorable prognostic effects of EDA weren't observed on neither overall survival nor disease recurrence among patients with esophageal cancer. We evaluated the contribution of EDA on recurrence in competing risks regression models. This modeling technique accounts for the effect of death without recurrence, which precludes the occurrence of a recurrence event. Competing risks regression provides a more conservative estimate of the effect relative to Cox regression or Kaplan-Meier models[29]. In competing risk regression analysis, EDA didn't reduce the CIR, compared with IVA.

There are some limitations that should be taken into consideration in interpretation of our results. As with all retrospective studies, this study was exposed to selection bias. Although we used propensity-score matching to compensate for some differences in baseline characteristics that may influence the outcomes after esophagectomy, intrinsic biases may still remain. Additionally, although in the minimally invasive esophagectomy (MIE) subgroup, EDA was also associated with lower incidence of pneumonia and anastomotic leakage, but neither reached statistical significance for the small sample size (data not shown). Further studies are still warranted to address this issue in the setting of MIE. Lastly, CRP, white blood cell and heart rate are non-specific markers of systematic inflammation, and the current study failed to measure specific inflammatory markers, such as interleukin-6, due to its retrospective nature.

In conclusion, the present propensity-matched analysis reveals that the use of EDA among patients undergoing esophagectomy, could attenuate the postoperative inflammatory response, decrease the incidence of pneumonia and anastomotic leakage. The main drawbacks of EDA were delayed urinary catheter removal and transient hemodynamic instability. Therefore, EDA remains to be considered as an important component of multimodal perioperative recovery after esophagectomy.

## Supporting Information

S1 File Patient data for statistical analyses. (XLS)

## References

[1] Biki B, Mascha E, Moriarty DC, Fitzpatrick JM, Sessler DI, Buggy DJ. Anesthetic technique for radical prostatectomy surgery affects cancer recurrence: a retrospective analysis. Anesthesiology. 2008;109(2):180–7. Epub 2008/07/24. 10.1097/ALN.0b013e31817f5b73 . 18648226

[2] Wuethrich PY, Hsu Schmitz SF, Kessler TM, Thalmann GN, Studer UE, Stueber F, et al Potential influence of the anesthetic technique used during open radical prostatectomy on prostate cancer-related outcome: a retrospective study. Anesthesiology. 2010;113(3):570–6. Epub 2010/08/05. 10.1097/ALN.0b013e3181e4f6ec . 20683253

[3] Hubner M, Blanc C, Roulin D, Winiker M, Gander S, Demartines N. Randomized clinical trial on epidural versus patient-controlled analgesia for laparoscopic colorectal surgery within an enhanced recovery pathway. Ann Surg. 2015;261(4):648–53. Epub 2014/08/15. 10.1097/SLA.0000000000000838 . 25119117

[4] Kamiyoshihara M, Nagashima T, Ibe T, Atsumi J, Shimizu K, Takeyoshi I. Is epidural analgesia necessary after video-assisted thoracoscopic lobectomy? Asian Cardiovasc Thorac Ann. 2010;18(5):464–8. Epub 2010/10/16. 10.1177/0218492310381817 . 20947601

[5] Rawal N. Epidural technique for postoperative pain: gold standard no more? Reg Anesth Pain Med. 2012;37(3):310–7. Epub 2012/04/26. 10.1097/AAP.0b013e31825735c6 . 22531384

[6] Luketich JD, Pennathur A, Awais O, Levy RM, Keeley S, Shende M, et al Outcomes after minimally invasive esophagectomy: review of over 1000 patients. Ann Surg. 2012;256(1):95–103. Epub 2012/06/07. 10.1097/SLA.0b013e3182590603 . 22668811 PMC4103614

[7] Wang H, Shen Y, Feng M, Zhang Y, Jiang W, Xu S, et al Outcomes, quality of life, and survival after esophagectomy for squamous cell carcinoma: A propensity score-matched comparison of operative approaches. J Thorac Cardiovasc Surg. 2015;149(4):1006–14; discussion 14–5 e4. Epub 2015/03/11. 10.1016/j.jtcvs.2014.12.063 . 25752374

[8] Law S, Wong K-H, Kwok K-F, Chu K-M, Wong J. Predictive Factors for Postoperative Pulmonary Complications and Mortality After Esophagectomy for Cancer. Ann Surg. 2004;240(5):791–800. 10.1097/01.sla.0000143123.24556.1c 15492560 PMC1356484

[9] Biere SS. Minimally invasive versus open oesophagectomy for patients with oesophageal cancer: a multicentre, open-label, randomised controlled trial. Lancet. 2012 10.1016/s0140-6736(12)60516-9 22552194

[10] Huang Q, Zhong J, Yang T, Li J, Luo K, Zheng Y, et al Impacts of anastomotic complications on the health-related quality of life after esophagectomy. J Surg Oncol. 2015;111(4):365–70. Epub 2014/11/25. 10.1002/jso.23837 . 25418352

[11] Rice TW, Blackstone EH, Rusch VW. 7th edition of the AJCC Cancer Staging Manual: esophagus and esophagogastric junction. Ann Surg Oncol. 2010;17(7):1721–4. Epub 2010/04/07. 10.1245/s10434-010-1024-1 . 20369299

[12] Yang H, Wang J, Huang Q, Zheng Y, Ela Bella A, Wang R, et al Intraoperative ultrasonography for the identification of thoracic recurrent laryngeal nerve lymph nodes in patients with esophageal cancer. Dis Esophagus. 2016;29(2):152–8. Epub 2015/01/22. 10.1111/dote.12318 . 25604726

[13] Huang Q, Luo K, Yang H, Wen J, Zhang S, Li J, et al Impact of alcohol consumption on survival in patients with esophageal carcinoma: a large cohort with long-term follow-up. Cancer Sci. 2014;105(12):1638–46. Epub 2014/10/08. 10.1111/cas.12552 . 25287715 PMC4317962

[14] Austin PC. Optimal caliper widths for propensity-score matching when estimating differences in means and differences in proportions in observational studies. Pharm Stat. 2011;10(2):150–61. Epub 2010/10/07. 10.1002/pst.433 20925139 PMC3120982

[15] Dignam JJ, Zhang Q, Kocherginsky M. The use and interpretation of competing risks regression models. Clin Cancer Res. 2012;18(8):2301–8. Epub 2012/01/28. 10.1158/1078-0432.CCR-11-2097 22282466 PMC3328633

[16] Gray RJ. A Class of K-Sample Tests for Comparing the Cumulative Incidence of a Competing Risk. The Annals of Statistics. 1988;16(3):1141–54.

[17] Hur C, Miller M, Kong CY, Dowling EC, Nattinger KJ, Dunn M, et al Trends in esophageal adenocarcinoma incidence and mortality. Cancer. 2013;119(6):1149–58. Epub 2013/01/11. 10.1002/cncr.27834 23303625 PMC3744155

[18] Rudin Å, Flisberg P, Johansson J, Walther B, Lundberg CJF. Thoracic Epidural Analgesia or Intravenous Morphine Analgesia After Thoracoabdominal Esophagectomy: A Prospective Follow-up of 201 Patients. J Cardiothorac Vasc Anesth. 2005;19(3):350–7. 10.1053/j.jvca.2005.03.013 16130063

[19] Saeki H, Ishimura H, Higashi H, Kitagawa D, Tanaka J, Maruyama R, et al Postoperative management using intensive patient-controlled epidural analgesia and early rehabilitation after an esophagectomy. Surg Today. 2009;39(6):476–80. Epub 2009/05/27. 10.1007/s00595-008-3924-2 . 19468802

[20] Michelet P, D'Journo XB, Roch A, Papazian L, Ragni J, Thomas P, et al Perioperative risk factors for anastomotic leakage after esophagectomy: influence of thoracic epidural analgesia. Chest. 2005;128(5):3461–6. Epub 2005/11/24. 10.1378/chest.128.5.3461 . 16304300

[21] Li LF, Liao SK, Ko YS, Lee CH, Quinn DA. Hyperoxia increases ventilator-induced lung injury via mitogen-activated protein kinases: a prospective, controlled animal experiment. Crit Care. 2007;11(1):R25 Epub 2007/02/24. 10.1186/cc5704 17316425 PMC2151853

[22] Fok M, Law SY, Wong J. Operable esophageal carcinoma: current results from Hong Kong. World J Surg. 1994;18(3):355–60. Epub 1994/05/01. . 8091775 10.1007/BF00316814

[23] Palomero Rodriguez MA, Suarez Gonzalo L, Villar Alvarez F, Varela Crespo C, Moreno Gomez Limon I, Criado Jimenez A. Thoracic epidural anesthesia decreases C-reactive protein levels in patients undergoing elective coronary artery bypass graft surgery with cardiopulmonary bypass. Minerva Anestesiol. 2008;74(11):619–26. Epub 2008/10/31. . 18971890

[24] Fares KM, Mohamed SA, Hamza HM, Sayed DM, Hetta DF. Effect of thoracic epidural analgesia on pro-inflammatory cytokines in patients subjected to protective lung ventilation during Ivor Lewis esophagectomy. Pain Physician. 2014;17(4):305–15. Epub 2014/07/24. . 25054390

[25] Ali M, Winter DC, Hanly AM, O'Hagan C, Keaveny J, Broe P. Prospective, randomized, controlled trial of thoracic epidural or patient-controlled opiate analgesia on perioperative quality of life. Br J Anaesth. 2010;104(3):292–7. Epub 2010/02/04. 10.1093/bja/aeq006 . 20124282

[26] Urschel JD. Esophagogastrostomy anastomotic leaks complicating esophagectomy: a review. Am J Surg. 1995;169(6):634–40. Epub 1995/06/01. . 7771633 10.1016/s0002-9610(99)80238-4

[27] Kusano C, Baba M, Takao S, Sane S, Shimada M, Shirao K, et al Oxygen delivery as a factor in the development of fatal postoperative complications after oesophagectomy. Br J Surg. 1997;84(2):252–7. Epub 1997/02/01. . 9052449

[28] Michelet P, Roch A, D'Journo XB, Blayac D, Barrau K, Papazian L, et al Effect of thoracic epidural analgesia on gastric blood flow after oesophagectomy. Acta Anaesthesiol Scand. 2007;51(5):587–94. Epub 2007/04/14. 10.1111/j.1399-6576.2007.01290.x . 17430321

[29] Lotan Y, Gupta A, Shariat SF, Palapattu GS, Vazina A, Karakiewicz PI, et al Lymphovascular invasion is independently associated with overall survival, cause-specific survival, and local and distant recurrence in patients with negative lymph nodes at radical cystectomy. Journal of clinical oncology: official journal of the American Society of Clinical Oncology. 2005;23(27):6533–9. Epub 2005/08/24. 10.1200/JCO.2005.05.516 . 16116151

